# Testicular Sertoli Cell Hormones in Differences in Sex Development

**DOI:** 10.3389/fendo.2022.919670

**Published:** 2022-07-14

**Authors:** Angela K. Lucas-Herald, Rod T. Mitchell

**Affiliations:** ^1^ Developmental Endocrinology Research Group, University of Glasgow, Glasgow, United Kingdom; ^2^ MRC Centre for Reproductive Health, The Queen’s Medical Research Institute, The University of Edinburgh, Edinburgh, United Kingdom; ^3^ Department of Paediatric Endocrinology, Royal Hospital for Children and Young People, Edinburgh, United Kingdom

**Keywords:** AMH, inhibin B, testes, spermatogenesis, Sertoli cell

## Abstract

The Sertoli cells of the testes play an essential role during gonadal development, in addition to supporting subsequent germ cell survival and spermatogenesis. Anti-Müllerian hormone (AMH) is a member of the TGF-β superfamily, which is secreted by immature Sertoli cells from the 8^th^ week of fetal gestation. lnhibin B is a glycoprotein, which is produced by the Sertoli cells from early in fetal development. In people with a Difference or Disorder of Sex Development (DSD), these hormones may be useful to determine the presence of testicular tissue and potential for spermatogenesis. However, fetal Sertoli cell development and function is often dysregulated in DSD conditions and altered production of Sertoli cell hormones may be detected throughout the life course in these individuals. As such this review will consider the role of AMH and inhibin B in individuals with DSD.

## Introduction

Gonadal development is a complex process, whereby the genital ridge is directed to develop into testes or ovaries. The testicular Sertoli cells play an essential role during gonadal development, in addition to supporting subsequent germ cell survival and spermatogenesis (1). Sertoli cells are responsible for the production of a variety of factors including hormones, binding proteins and signalling molecules that regulate testicular development and function throughout life. Anti-Müllerian hormone (AMH) and inhibin B are hormones that play a role in sex development during fetal life, as well as regulation of spermatogenesis in adulthood. Differences/Disorders of Sex Development (DSD) are a heterogeneous group of conditions with a wide range of aetiologies and clinical features. The presence of measurable AMH and inhibin B in infants with DSD suggests the presence of testicular tissue and indicates Sertoli cell function. However, in people with a DSD, fetal Sertoli cell development and function can be dysregulated. As both of these hormones can be useful clinically, this review will focus on the role of testicular AMH and inhibin B in individuals with DSD.

## Pathways of Typical Sex Development and Testicular Cell Differentiation

In human embryos, gonadal precursors are present from 32 days post conception and the gonads are bipotential until 6 weeks of gestation (2). As the testicular cords are established at 6-7 weeks post fertilisation, the Sertoli and interstitial cells (including Leydig-lineage cells) originate from common gonadal progenitors and subsequently differentiate (3, 4)

Sry, sex determination region on Y chromosome is largely responsible for testis differentiation. The Sry gene is expressed in pre-Sertoli cells at 7 weeks in the XY gonad and encodes a high mobility group (HMG) box transcription factor, which binds to specific target sequences in DNA, resulting in DNA bending (5). The expression of Sry is initiated by multiple transcription factors including GATA4/FOG2/NR5A11/WT1, resulting in induction of SOX9 expression. This is further augmented by the synergistic actions of Sry and NR5A1, leading to definitive Sertoli cell differentiation (6, 7).

Much of the data relating to sex differentiation in mammals is derived from mouse studies. However, studies using human fetal tissues provide support for these mechanisms also being important for sex development in humans. Mamsen et al. undertook gene expression analysis of key genes associated with gonadal development in 67 human first trimester fetuses obtained during elective termination of pregnancy. This study demonstrated that in the bipotential gonad, WT1 and NR5A1 were highly expressed, although concentrations of WT1 decreased over time. SOX9 gene expression increased to a peak at day 48. AMH was detected in Sertoli cells from 48 days. SRY expression peaked at 44 days post conception and then decreased to basal levels at day 60 (8). That said, SRY expression has also been identified in 46,XY gonads up to 18 weeks gestation in human embryonic and fetal tissue (9).

Once formed, Sertoli cells induce the development of fetal Leydig cells, *via* a *hedgehog* signalling pathway (10). At 8-9 weeks of development, the Leydig cells start to produce androgens and insulin-like factor 3 (INSL3) (11). Masculinisation of the indifferent external genitalia is induced by testosterone produced by the Leydig cells between weeks 9-20 of gestation (12, 13). Testicular descent is primarily under the control of the Leydig cells *via* the actions of INSL3 and testosterone. INSL3 regulates the first phase of testicular descent, acting *via* cyclic AMP with downstream effects *via* Wnt, β-catenin and BMP, causing the gubernaculum to swell, dilating the future inguinal canal and holding the testis close to the groin as the fetal abdomen enlarges between weeks 8-15 of gestation. The second stage of testis descent from the abdomen to the inguino-scrotal region occurs from approximately 25 weeks of gestation due to shortening of the gubernaculum cord and is dependent on the presence of sufficient exposure to androgen (14).

## AMH – Secretion and Regulation

AMH, previously known as Müllerian Inhibiting Substance (MIS), is a member of the TGF-β superfamily. It is a 140-kDa dimer glycoprotein, which is secreted by immature Sertoli cells from the 8^th^ week of fetal gestation (15).

The AMH gene is located on chromosome 19 (16). Gonadotrophin-independent transcription is upregulated by SOX9, NR5A1, GATA4, WT1, AP-1 and AP-2. Late in fetal life and after birth, AMH transcription falls under the control of FSH *via* the adenyl-cyclase cyclic AMP (cAMP) pathway (17). Increased testicular AMH production in response to FSH activates protein kinase A (PKA)-mediated induction of SOX9, SF1, NFkB and AP-2, which bind to specific response elements on the AMH promoter (17, 18). FSH stimulation upregulates AMH transcription by phosphorylating the transcription factors binding to the promoter (19), an effect which is downregulated by testosterone (15).

Immunohistochemical labelling of testes has shown that the AR is expressed weakly in 2-15% of Sertoli cells from approximately the age of 5 months until the age of 4 years resulting in a physiological Sertoli cell androgen insensitivity during fetal and early postnatal life, which may protect the testes from premature Sertoli cell maturation (20). Expression progressively increases thereafter such that 90% of boys had high levels of AR expression from the age of 8 years (20). However, the AMH promoter does not have androgen response elements, and as such the androgen receptor must signal indirectly through SF-1 response elements (21).

## Inhibin B – Secretion and Regulation

lnhibin B is a glycoprotein, secreted by Sertoli cells, which consists of α- and β-subunits. Most of its mechanism of action is *via* antagonism of activins on the activin type I and II receptors but there are some cells with specific inhibin-binding molecules, such as betaglycan (22). Recent genome wide association studies (GWAS) have demonstrated that LRRIQ1 and TSPAN19, two genes located on chromosome 12 may affect inhibin B production (23). Plasma inhibin B measurements reflect both Sertoli cell number and status of spermatogenesis (24). Inhibin B has a complex association with the hypothalamic-pituitary-gonadal (HPG) axis and FSH, with an initially positive association at around 3-6 months of age and prior to the onset of puberty, followed by a negative feedback loop (25).

## Testicular Hormone Production in Fetal and Early Postnatal Life

**Figure 1:**
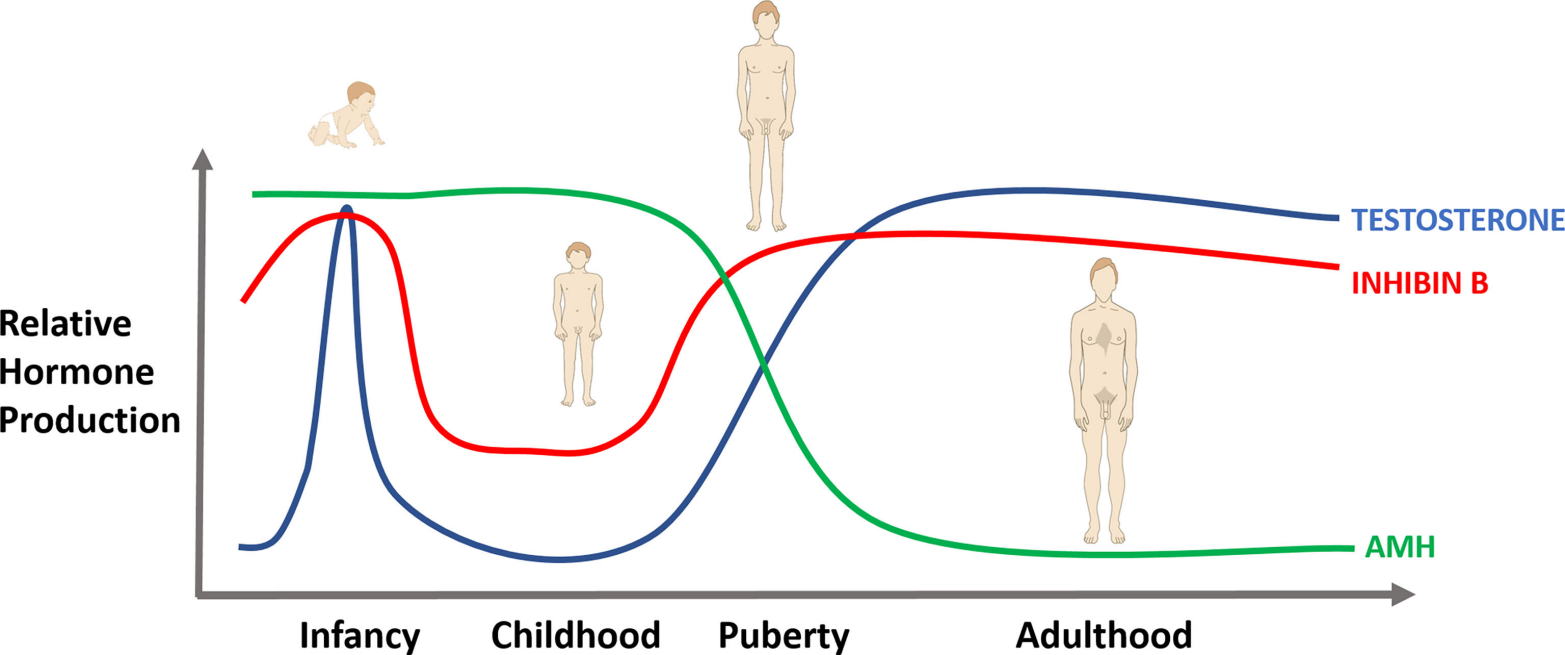
Levels of testosterone, AMH and inhibin B from fetal life to adulthood.

Testosterone production by the human fetal testis begins around 8 weeks gestation, with a peak between 14-17 weeks and then a sharp decline, so that in late pregnancy the serum concentration of testosterone is similar in male and female foetuses (**Figure 1**) (2, 26). Gonadotrophins are not required to initiate steroid synthesis during this time, but levels of testosterone are closely correlated with human chorionic gonadotrophin (hCG) levels during the early gestational period (2). Levels of testosterone and gonadotrophins are low towards the end of pregnancy and at birth before increasing in the early postnatal period, the so-called ‘mini-puberty’, and can be used as a window to assess the activity of the HPG axis in the first few months of postnatal life. However, they are of limited use to assess testicular function in the prepubertal boy, because of the relative inactivity of the axis during childhood.

AMH is produced at high levels in early fetal life, but is not measurable in amniotic fluid (27, 28). As the testes differentiate during the 7^th^ week of gestation, Sertoli cells start producing AMH, which binds to the specific AMH Receptor Type II (AMHR2) on the Müllerian ducts, resulting in their regression before week 10 (29). AMH levels rise progressively from this point and then decline in the second year of life (**Figure 1**). AMH then remains stable until puberty, at which point it declines to adult levels, which are 3-4% of those in infancy (30). AMH is therefore an excellent marker of Sertoli cell function in infancy and early childhood, but its use becomes more difficult to interpret in adolescents with lower levels, as at this stage, reduction in AMH is an indicator of pubertal terminal differentiation and failure of the Sertoli cells (31). Serum levels of AMH are 50-fold lower in girls than boys at birth (32) and as such, it is a useful marker to confirm the presence of testicular tissue. No extragonadal sites of AMH production have been reported to date. Given that levels do vary with age, care must be taken with interpretation of AMH to ensure the correct reference range is used.

Inhibin B has been detected from 14-16 weeks gestation (33). and has been detected in umbilical cord samples in boys but not girls (34). Between 3-6 months of age, inhibin B rises in line with FSH and a concurrent increase in Sertoli cell number (35) (**Figure 1**). Levels persist after testosterone, LH and FSH start to fall (25). Levels then remain consistent until approximately 8 years of age, peaking at around age 17 before a slow decline to adult levels (36). Inhibin B is therefore a useful indicator of spermatogenesis and Sertoli cell function in adults, when AMH is no longer measurable.

## The Role of Fetal Sertoli Cell Hormones in DSD

Differences in AMH and inhibin B can be seen in many forms of DSD. These changes have been reviewed extensively elsewhere (37) but this review will discuss some specific DSD conditions.

### Sex Chromosome DSD

Klinefelter syndrome, 47,XXY, is the most common sex chromosome anomaly, affecting around 1 in 660 live male births (38). Men with this condition have hypergonadotrophic hypogonadism, as well as being 6 times more likely to have cryptorchidism, which will also impact on Sertoli cell function (39). A study by Aksglaede et al., measured AMH levels in a cohort of 95 men with non-mosaic Klinefelter syndrome and demonstrated that levels remained within the population reference range in infancy, childhood and into adolescence (40). In 47,XXY infants, levels of inhibin B have also been shown to remain in the normal reference range for age (41). Based on these findings, it is presumed that fetal levels of AMH and inhibin B will also be similar between boys with Klinefelter syndrome and controls. However, levels of inhibin B do reduce significantly with age once adulthood is attained, in addition to low AMH levels reflecting the progressive degeneration of the seminiferous tubules (40).

A recent study by Spaziani et al. (10.1007/s40618-020-01281-x) has also found that inhibin B and AMH can be high in early childhood and mini-puberty, suggesting that further research is required in this cohort of patients to confirm expected biochemical findings.

### 46,XX DSD

#### Ovotesticular DSD

About 65% of those with ovotesticular DSD have a 46,XX chromosome complement and the ability of the ovotestes to function will be variable. As such, AMH and inhibin B levels in fetal life will vary from being high for female but low for male to normal for male, depending on the clinical phenotype and assays used. Generally, in fully virilised 46,XX males, AMH, inhibin B and testosterone will be within the normal male range in childhood but the germ cells will fail to undergo complete meiosis and undergo apoptosis at puberty, resulting in low testicular volumes (15). In 46,XX children with atypical genitalia, AMH levels above the normal female range are highly suggestive of ovotesticular DSD and exclude the differential diagnoses of congenital adrenal hyperplasia, aromatase defects or virilising tumours.

SOX9 gene variants have been associated with ovotesticular DSD and skeletal dysplasias (42). The SRY gene upregulates SOX9 expression and once levels of SOX9 have reached a critical threshold, several positive regulatory loops are initiated, including autoregulation of SOX9 expression and formation of feed-forward loops *via* FGF9 or PGD2 signalling, which are required for the maintenance and sustained function of Sertoli cells (43). During testicular development, SOX9 functions by regulating the production of AMH from Sertoli cells, and possibly by repressing genes involved in ovarian development such as *Wnt4* and *Foxl2* (44). Studies regarding boys with SOX9 variants, report AMH levels, which are low to low normal (45, 46), with insufficient data regarding inhibin B levels to date.

### 46,XY DSD

#### Disorders of AMH and AMH Receptor Defects

From around 7 weeks gestation, the AMH gene is activated by SF1 in Sertoli cells. This leads to the regression of Müllerian structures in the developing male fetus (47). Where there is a mutation in the AMH gene, or in AMHR2, Persistent Müllerian Duct Syndrome (PMDS) occurs. Boys most commonly present with cryptorchidism, inguinal herniae and later infertility. Orchidopexy can be challenging, likely because the testes often have an excessively elongated gubernacular cord, potentially due to the mechanical effects of the retained uterus, or because AMH has an effect on shortening the gubernacular cord (14). A longitudinal study of 157 men with PMDS demonstrated that testicular malignant transformation occurs in 33% of individuals with PMDS (48). Malignancy has been reported in cases of PMDS with cryptorchidism and transverse testicular ectopia but has also been seen in normal testis in patients with PMDS, suggesting that the mechanism for malignancy is not just related to mechanical cryptorchidism (49). In approximately 8% of cases, malignant transformation of the Müllerian remnants can also occur, particularly after puberty, although again the mechanism for this is unknown (50). Treatment is primarily surgical, with excision of Müllerian remnants to allow for orchidopexy. Leydig cell function is typically normal but AMH levels will be low or undetectable in those with an AMH gene variant. In contrast, those with a mutation in AMHR2 will have normal-for-age AMH levels (48).

#### Disorders of Gonadal Development

Approximately 15% of all cases of 46,XY complete gonadal dysgenesis (CGD) result from a deletion in SRY, with the majority being located within the HMG domain (51). AMH and inhibin B levels will be low/undetectable in individuals with CGD, with the absolute level correlating to the amount of gonadal tissue and number of functioning Sertoli cells present. As such individuals with partial gonadal dysgenesis (PGD) tend to have higher levels than those with CGD (52).

#### Disorders of Androgen Action

Complete and partial androgen insensitivity syndrome (CAIS and PAIS respectively) are characterised by mutations in the AR gene. Testis differentiation and development, as well as gene expression patterns of AMH and AMHR are independent of AR action up to the second trimester of pregnancy. This has been confirmed by post-mortem examinations of fetuses with AR defects and expression of AMH, AMH2 and testicular differentiation markers (53). During childhood, AMH levels are usually within the normal range in boys with PAIS. By puberty, testicular AMH increases in young people with AIS, in tandem with FSH and oestradiol levels. The expression of Oestrogen Receptor α (ERα) has been confirmed in Sertoli cells from patients with CAIS (54). AMH may be a useful tool to distinguish CAIS and PAIS. A recent study of 29 AIS patients under the age of 11 years reported lower AMH levels in individuals with CAIS compared to PAIS, although still within the normal range for men (55).

Inhibin B levels have been measured in different cohorts of boys with PAIS, with median levels being lower in these boys compared to controls at all ages but usually still within the normal range and higher than in other forms of XY DSD (56, 57). No statistically significant differences are reported in inhibin B between individuals with CAIS and PAIS (55).

#### Disorders of Androgen Synthesis

Studies of inhibin B and AMH in boys with 5α-reductase type 2 deficiency (5ARD2) compared to other DSD conditions and controls have found that boys with 5ARD2 had lower levels of both hormones compared to controls (56).

#### Non-Specific Disorders of Undermasculinisation

Cryptorchidism occurs due to failure of descent of the testes and is a common congenital disorder, reported to affect up to 9% of male infants in some populations (58). Boys with both unilateral and bilateral cryptorchidism have been demonstrated to have lower AMH levels than control boys, indicating testicular dysfunction in childhood. Postnatal maturation of Sertoli cells is altered in cryptorchidism and a study of 40 infants with cryptorchidism aged 4-35 months showed strong positive correlations between inhibin B, LH and FSH with Sertoli cell number (59)

A recent retrospective study of 310 prepubertal boys with cryptorchidism confirmed that whilst low AMH was prevalent in boys with both unilateral and bilateral cryptorchidism, lower levels were seen in boys with bilateral undescended testes (60). In addition, in terms of treatment, testicular descent was more likely to be successful in response to treatment with hCG in those with a higher AMH at baseline, suggesting this may be a useful predictive marker when counselling families regarding the advantages and disadvantages of hormonal versus surgical management (60).

In boys with undermasculinisation, hCG stimulation may be used to assess Leydig cell function in tandem with an AMH measurement to assess Sertoli cell function. In 138 children with a non-specific XY DSD, a normal AMH was predictive of a normal testosterone response to hCG, suggesting that where Sertoli cell function is preserved, Leydig cell function is also likely to be (61). Of the 138 boys in the study cohort, 53 (38%) had combined genital anomalies; 47 (34%) had isolated bilateral undescended testes and 29 (21%) had isolated proximal hypospadias. Boys with isolated hypospadias had a higher AMH and higher testosterone after stimulation with human chorionic gonadotrophin (hCG) compared to children with isolated bilateral undescended testes (p=0.0001) or children with combined anomalies including undescended testes (p<0.0001) (61). Children with undescended testes but no other genital anomalies had the lowest AMH and amongst those with bilateral undescended testes, children with impalpable testes had a lower median AMH than children with inguinal testes (470 (1.5, 1926) vs 832 (72, 2280) pmol/l (p=0.04) (61).

Testicular dysgenesis syndrome (TDS) is a term used to describe a group of associated male reproductive disorders that arise as a result of impaired androgen production or action during a critical period of fetal testicular development (62). The reduced androgen exposure is associated with cryptorchidism, hypospadias, testicular germ cell tumours and subfertility. Dysgenesis (often focal) within the testis is a frequent finding in men with these disorders, which includes undifferentiated Sertoli cells (63, 64). Animal models have been used to study Sertoli cell development and maturation in this cohort. Pregnancy exposure to di(n-Butyl) phthtalate in rats produces a similar phenotype and demonstrate altered Sertoli cell maturation, with a worse phenotype seen in cryptorchid testes compared to scrotal testes (65).

In anorchia, AMH and inhibin B levels are both undetectable, usually in combination with raised FSH levels (66).

#### Specific Gene Variants

NR5A1 (also known as Steroidogenic Factor 1 or SF1) is a nuclear receptor transcription factor whose expression commences in the coelomic epithelium and continues in steroidogenic cells. Its expression has been demonstrated in the bipotential gonad from 32 days post conception and following testis determination (around 42 days onward), its expression is maintained in the somatic cells of the early testis, which suggests it may play a role in supporting SOX9 expression (67). It is also known to activate the expression of AMH in Sertoli cells from around 7 weeks gestation, resulting in the regression of Müllerian structures in the developing male foetus and is responsible for activating the expression of steroidogenic enzymes from 8 weeks gestation, resulting in the androgenisation of the external genitalia (9). AMH and inhibin B levels in individuals with NR5A1 gene variants have been reported as normal to low-normal (68, 69).

Early studies of simulated FSH deficiency *via* administration of hCG demonstrated that FSH is required for normal spermatogenesis, although sperm production was not entirely suppressed in the absence of FSH (70). Babies born with FSH receptor mutations, have Sertoli cell hypoplasia and small testis resulting in low spermatogenesis and low inhibin B in adulthood. Müllerian structures do regress however suggesting AMH levels are likely to be normal in early fetal life (71).

GATA-binding protein 4 (GATA4) is a transcription factor which is known to be involved in the development of some forms of congenital heart disease (72). Studies with GATA4 mutations have demonstrated its likely involvement in gonadal development in conjunction with its cofactor, Friend of GATA2 (FOG2) (73). These genes are upstream of SRY and when mutated cause significant reductions in SRY expression [Sekido and Lovell-Badge, 2013]. In a family of individuals with mutations in GATA4, 46,XY DSD and congenital heart disease, AMH levels were consistently low and functional analysis demonstrated that mutations in GATA4 may reduce the action of the AMH promoter (74).

#### Hypogonadotrophic Hypogonadism

In cases of congenital hypogonadotrophic hypogonadism, deficient LH and FSH does not affect Müllerian regression and early sex development, but does impair genital development, which is dependent on testosterone from mid to late pregnancy. This may result in small testes as a result of FSH deficiency and micropenis +/- cryptorchidism arising from LH deficiency (75). A study of 8 men with hypogonadotrophic hypogonadism (4 with Kallmann’s syndrome, 4 idiopathic), demonstrated that AMH was high for age, because serum testosterone remained low and therefore did not downregulate AMH. Treatment with recombinant human FSH increased serum AMH, Further treatment with hCG increased testosterone and reduced AMH and inhibin B (76).

### Current Research Gaps

Overall, whilst many studies have considered AMH concentrations as an indicator of testicular development in children and young people with DSD, very few have focussed on inhibin B. Therefore, the use of Inhibin B as a marker of Sertoli cell function should be a research priority in future years, particularly when assessing adolescents with DSD, in whom AMH levels are more difficult to interpret.

In young children with non-specific 46,XY disorders of undermasculinisation, AMH and inhibin B levels both show good correlation with post-hCG testosterone (61, 77), potentially obviating the need for a stimulated test. hCG stimulation tests are invasive for the child and logistically difficult for the healthcare team and family and as such, studies assessing the clinical utility of AMH or inhibin B levels as an alternative to hCG testing to assess gonadal function will be invaluable.

### Summary and Conclusions

To summarize, fetal Sertoli cell hormones are crucial for normal sex development. In particular, AMH is responsible for regression of Müllerian structures. Both AMH and inhibin B represent useful biomarkers in children with DSD conditions, as they allow for confirmation of the presence of testicular tissue, as well as monitoring of gonadal function. However, their secretion is age-dependent, requiring specific reference ranges and reliable assays if they are to be used in clinical practice. Serum levels of both, in conjunction with those of androgens and gonadotrophins, can, however, be helpful in the diagnosis of DSD conditions, with specific patterns being more likely to be seen in certain disorders.

## Author Contributions

Both authors contributed sugnificantly to the conception and writing of this manuscript. Both authors agreed on the final version.

## Funding

AL-H is funded by the NES/CSO Joint Clinical Lectureship scheme. RM is supported by UKRI Future Leaders Fellowship (MR/S017151/1). The MRC Centre for Reproductive Health at the University of Edinburgh is supported by MRC (MR/N022556/1).

## Conflict of Interest

The authors declare that the research was conducted in the absence of any commercial or financial relationships that could be construed as a potential conflict of interest.

## Publisher’s Note

All claims expressed in this article are solely those of the authors and do not necessarily represent those of their affiliated organizations, or those of the publisher, the editors and the reviewers. Any product that may be evaluated in this article, or claim that may be made by its manufacturer, is not guaranteed or endorsed by the publisher.

## References

[1] França LR Hess RA Dufour JM Hofmann M Griswold MD. The Sertoli Cell: One Hundred Fifty Years of Beauty and Plasticity. Andrology (2016) 4:189–212. doi: 10.1111/andr.12165 26846984 PMC5461925

[2] Rey R Josso N Racine C. Sexual Differentiation. In: Feingold KR Anawalt B Boyce A Chrousos G de Herder WW Dhatariya K Endotext. South Dartmouth (MA): MDText. com, Inc. (2016) 2000:905232. 25905232

[3] Guo J Sosa E Chitiashvili T Nie X Rojas EJ Oliver E. Single-Cell Analysis of the Developing Human Testis Reveals Somatic Niche Cell Specification and Fetal Germline Stem Cell Establishment. Cell Stem Cell (2021) 28:764–78.e4. doi: 10.1016/j.stem.2020.12.004 33453151 PMC8026516

[4] Nef S Stévant I Greenfield A. Chapter Six - Characterizing the Bipotential Mammalian Gonad. In: Capel B, editor. Curr Top Dev Biol. Academic Press (2019) 134:167–94. 10.1016/bs.ctdb.2019.01.002 30999975

[5] Okashita N Tachibana M. Transcriptional Regulation of the Y-Linked Mammalian Testis-Determining Gene SRY. Sexual Dev (2021) 15:351–9. doi: 10.1159/000519217 34583357

[6] Zhao L Koopman P. SRY Protein Function in Sex Determination: Thinking Outside the Box. Chromosome Res (2012) 20:153–62. doi: 10.1007/s10577-011-9256-x 22161124

[7] Sekido R Lovell-Badge R. Sex Determination Involves Synergistic Action of SRY and SF1 on a Specific Sox9 Enhancer. Nature (2008) 453:930–4. doi: 10.1038/nature06944 18454134

[8] Mamsen LS Ernst EH Borup R Larsen A Olesen RH Ernst E. Temporal Expression Pattern of Genes During the Period of Sex Differentiation in Human Embryonic Gonads. Sci Rep (2017) 7:15961. doi: 10.1038/s41598-017-15931-3 29162857 PMC5698446

[9] Hanley N Ball S Clement-Jones M Hagan D Strachan T Lindsay S. Expression of Steroidogenic Factor 1 and Wilms' Tumour 1 During Early Human Gonadal Development and Sex Determination. Mech Dev (1999) 87:175–80. doi: 10.1016/S0925-4773(99)00123-9 10495282

[10] Yao HH Whoriskey W Capel B. Desert Hedgehog/Patched 1 Signaling Specifies Fetal Leydig Cell Fate in Testis Organogenesis. Genes Dev (2002) 16:1433–40. doi: 10.1101/gad.981202 PMC186321 12050120

[11] Barsoum IB Bingham NC Parker KL Jorgensen JS Yao HH-C. Activation of the Hedgehog Pathway in the Mouse Fetal Ovary Leads to Ectopic Appearance of Fetal Leydig Cells and Female Pseudohermaphroditism. Dev Biol (2009) 329:96–103. doi: 10.1016/j.ydbio.2009.02.025 19268447 PMC2673990

[12] Moore K Persaud T Torchia M. The Developing Human: Clinically Orientated Embryology. Philadelphia, US: Elsevier (2016).

[13] Welsh M Suzuki H Yamada G. The Masculinization Programming Window. Endocr Dev (2014) 27:17–27. doi: 10.1159/000363609 25247641

[14] Hutson JM. Undescended Testis: The Underlying Mechanisms and the Effects on Germ Cells That Cause Infertility and Cancer. J Pediatr Surg (2013) 48:903–8. doi: 10.1016/j.jpedsurg.2013.02.001 23701757

[15] Edelsztein NY Grinspon RP Schteingart HF Rey RA. Anti-Müllerian Hormone as a Marker of Steroid and Gonadotropin Action in the Testis of Children and Adolescents With Disorders of the Gonadal Axis. Int J Pediatr Endocrinol (2016) 2016:20. doi: 10.1186/s13633-016-0038-2 27799946 PMC5084469

[16] Cohen-Haguenauer O Picard J Mattei M-G Serero S Van Cong N De Tand M-F. Mapping of the Gene for Anti-Müllerian Hormone to the Short Arm of Human Chromosome 19. Cytogenet Genome Res (1987) 44:2–6. doi: 10.1159/000132332 3028714

[17] Lasala C Schteingart HF Arouche N Bedecarrás P Grinspon RP Picard J-Y. SOX9 and SF1 are Involved in Cyclic AMP-Mediated Upregulation of Anti-Müllerian Gene Expression in the Testicular Prepubertal Sertoli Cell Line SMAT1. Am J Physiol Endocrinol Metab (2011) 301:E539–47. doi: 10.1152/ajpendo.00187.2011 21693691

[18] Josso N Rey RA. What Does AMH Tell Us in Pediatric Disorders of Sex Development? Front Endocrinol (2020) 11:619. doi: 10.3389/fendo.2020.00619 PMC7506080 33013698

[19] Josso N Picard J-Y. Genetics of Anti-Müllerian Hormone and Its Signaling Pathway. Best Pract Res Clin Endocrinol Metab (2022) 36:101634. doi: 10.1016/j.beem.2022.101634 35249805

[20] Chemes HE Rey RA Nistal M Regadera J Musse M Gonzaílez-Peramato P. Physiological Androgen Insensitivity of the Fetal, Neonatal, and Early Infantile Testis Is Explained by the Ontogeny of the Androgen Receptor Expression in Sertoli Cells. J Clin Endocrinol Metab (2008) 93:4408–12. doi: 10.1210/jc.2008-0915 18713818

[21] Edelsztein NY Racine C di Clemente N Schteingart HF Rey RA. Androgens Downregulate Anti-Müllerian Hormone Promoter Activity in the Sertoli Cell Through the Androgen Receptor and Intact Steroidogenic Factor 1 Sites. Biol Reprod (2018) 99:1303–12. doi: 10.1093/biolre/ioy152 29985989

[22] Luisi S Florio P Reis FM Petraglia F. Inhibins in Female and Male Reproductive Physiology: Role in Gametogenesis, Conception, Implantation and Early Pregnancy. Hum Reprod Update (2005) 11:123–35. doi: 10.1093/humupd/dmh057 15618291

[23] Sato Y Tajima A Kiguchi M Kogusuri S Fujii A Sato T. Genome-Wide Association Study of Semen Volume, Sperm Concentration, Testis Size, and Plasma Inhibin B Levels. J Hum Genet (2020) 65:683–91. doi: 10.1038/s10038-020-0757-3 32341457

[24] Sharpe RM Turner KJ McKinnell C Groome NP Atanassova N Millar MR. Inhibin B Levels in Plasma of the Male Rat From Birth to Adulthood: Effect of Experimental Manipulation of Sertoli Cell Number. J Androl (1999) 20:94–101. doi: 10.1002/j.1939-4640.1999.tb02501.x 10100479

[25] Anderson RA Sharpe RM. Regulation of Inhibin Production in the Human Male and Its Clinical Applications. Int J Androl (2000) 23:136–44. doi: 10.1046/j.1365-2605.2000.00229.x 10844538

[26] Scott HM Mason JI Sharpe RM. Steroidogenesis in the Fetal Testis and Its Susceptibility to Disruption by Exogenous Compounds. Endocr Rev (2009) 30:883–925. doi: 10.1210/er.2009-0016 19887492

[27] Josso N Lamarre I Picard J-Y Berta P Davies N Morichon N. Anti-Müllerian Hormone in Early Human Development. Early Hum Dev (1993) 33:91–9. doi: 10.1016/0378-3782(93)90204-8 8055780

[28] Kuijper EAM Ket JCF Caanen MR Lambalk CB. Reproductive Hormone Concentrations in Pregnancy and Neonates: A Systematic Review. Reprod BioMed Online (2013) 27:33–63. doi: 10.1016/j.rbmo.2013.03.009 23669015

[29] Brunello FG Rey RA. AMH and AMHR2 Involvement in Congenital Disorders of Sex Development. Sexual Dev (2021) 31:1–9. doi: 10.1159/000518273 34515230

[30] Aksglaede L Sørensen K Boas M Mouritsen A Hagen CP Jensen RB. Changes in Anti-Müllerian Hormone (AMH) Throughout the Life Span: A Population-Based Study of 1027 Healthy Males From Birth (Cord Blood) to the Age of 69 Years. J Clin Endocrinol Metab (2010) 95:5357–64. doi: 10.1210/jc.2010-1207 20843948

[31] Chojnacka K Zarzycka M Mruk DD. Biology of the Sertoli Cell in the Fetal, Pubertal, and Adult Mammalian Testis. Mol Mech Cell Differ Gonad Dev (2016) 58:225–51. doi: 10.1007/978-3-319-31973-5_9 27300181

[32] Bergadá I Milani C Bedecarrás P Andreone L Ropelato MG Gottlieb S. Time Course of the Serum Gonadotropin Surge, Inhibins, and Anti-Mullerian Hormone in Normal Newborn Males During the First Month of Life. J Clin Endocrinol Metab (2006) 91:4092–8. doi: 10.1210/jc.2006-1079 16849404

[33] Muttukrishna S Jauniaux E McGarrigle H Groome N Rodeck C. In-Vivo Concentrations of Inhibins, Activin A and Follistatin in Human Early Pregnancy. Reprod Biomed Online (2004) 8:712–9. doi: 10.1016/S1472-6483(10)61653-7 15169590

[34] De Schepper J Verlinde F Cortvrindt R Callewaert M Smitz J. Serum Inhibin B in Normal Term-Born Male and Female Neonates During the First Week of Life. Eur J Pediatr (2000) 159:465–9. doi: 10.1007/s004310051309 10867856

[35] Cortes D Müller J Skakkebaek NE. Proliferation of Sertoli Cells During Development of the Human Testis Assessed by Stereological Methods. Int J Androl (1987) 10:589–96. doi: 10.1111/j.1365-2605.1987.tb00358.x 3654012

[36] Kelsey TW Miles A Mitchell RT Anderson RA Wallace WHB. A Normative Model of Serum Inhibin B in Young Males. PLoS One (2016) 11:e0153843. doi: 10.1371/journal.pone.0153843 27077369 PMC4831823

[37] Johannsen TH Andersson AM Ahmed SF de Rijke YB Greaves RF Hartmann MF. Peptide hormone analysis in diagnosis and treatment of Differences of Sex Development: joint position paper of EU COST Action 'DSDnet' and European Reference Network on Rare Endocrine Conditions. Eur J Endocrinol. (2020) 182(6):P1–P15. doi: 10.1530/EJE-19-0831 10.1530/EJE-19-0831 32268295

[38] Bojesen A Juul S Gravholt CH. Prenatal and Postnatal Prevalence of Klinefelter Syndrome: A National Registry Study. J Clin Endocrinol Metab (2003) 88:622–6. doi: 10.1210/jc.2002-021491 12574191

[39] Bojesen A Juul S Birkebæk NH Gravholt CH. Morbidity in Klinefelter Syndrome: A Danish Register Study Based on Hospital Discharge Diagnoses. J Clin Endocrinol Metab (2006) 91:1254–60. doi: 10.1210/jc.2005-0697 16394093

[40] Aksglaede L Christiansen P Sørensen K Boas M Linneberg A Main KM. Serum Concentrations of Anti-Müllerian Hormone (AMH) in 95 Patients With Klinefelter Syndrome With or Without Cryptorchidism. Acta Paediatr (2011) 100:839–45. doi: 10.1111/j.1651-2227.2011.02148.x 21251056

[41] Cabrol S Ross JL Fennoy I Bouvattier C Roger M Lahlou N. Assessment of Leydig and Sertoli Cell Functions in Infants With Nonmosaic Klinefelter Syndrome: Insulin-Like Peptide 3 Levels Are Normal and Positively Correlated With LH Levels. J Clin Endocrinol Metab (2011) 96:E746–53. doi: 10.1210/jc.2010-2103 PMC5393421 21307139

[42] Ahmed SF Bashamboo A Lucas-Herald A McElreavey K. Understanding the Genetic Aetiology in Patients With XY DSD. Br Med Bull (2013) 106:67–89. doi: 10.1093/bmb/ldt008 23529942

[43] Wilhelm D Palmer S Koopman P. Sex Determination and Gonadal Development in Mammals. Physiol Rev (2007) 87(1):1–28. doi: 10.1152/physrev.00009.2006 17237341

[44] Schlessinger D Garcia-Ortiz JE Forabosco A Uda M Crisponi L Pelosi E. Determination and Stability of Gonadal Sex. J Androl (2010) 31:16–25. doi: 10.2164/jandrol.109.008201 19875493 PMC2882171

[45] Lee GM Ko JM Shin CH Yang SW. A Korean Boy With 46,XX Testicular Disorder of Sex Development Caused by SOX9 Duplication. Ann Pediatr Endocrinol Metab (2014) 19:108–12. doi: 10.6065/apem.2014.19.2.108 PMC4114044 25077096

[46] Benko S Gordon CT Mallet D Sreenivasan R Thauvin-Robinet C Brendehaug A. Disruption of a Long Distance Regulatory Region Upstream of &Lt;Em<SOX9&lt;/em< in Isolated Disorders of Sex Development. J Med Genet (2011) 48:825. doi: 10.1136/jmedgenet-2011-100255 22051515

[47] Biason-Lauber A. The Battle of the Sexes: Human Sex Development and its Disorders. Mol Mech Cell Differ Gonad Dev (2016) 58:337–82. doi: 10.1007/978-3-319-31973-5_13 27300185

[48] Picard JY Cate RL Racine C Josso N. The Persistent Müllerian Duct Syndrome: An Update Based Upon a Personal Experience of 157 Cases. Sexual Dev (2017) 11:109–25. doi: 10.1159/000475516 28528332

[49] Yang C Chen H Huang Y Li P Tian R Li Z. Transverse Testicular Ectopia Associated With Persistent Mullerian Duct Syndrome in Infertile Male: Two Case Reports and Literature Review. Trans Androl Urol (2021) 10:512–9. doi: 10.21037/tau-20-888 PMC7844511 33532339

[50] Farikullah J Ehtisham S Nappo S Patel L Hennayake S. Persistent Müllerian Duct Syndrome: Lessons Learned From Managing a Series of Eight Patients Over a 10-Year Period and Review of Literature Regarding Malignant Risk From the Müllerian Remnants. BJU Int (2012) 110:E1084–9. doi: 10.1111/j.1464-410X.2012.11184.x 22540537

[51] Rocha VBC Guerra-Júnior G Marques-de-Faria AP de Mello MP Maciel-Guerra AT. Complete Gonadal Dysgenesis in Clinical Practice: The 46, XY Karyotype Accounts for More Than One Third of Cases. Ferti Steril (2011) 96:1431–4. doi: 10.1016/j.fertnstert.2011.09.009 21982289

[52] Bastian C Muller J-B Lortat-Jacob S Nihoul-Fékété C Bignon-Topalovic J McElreavey K. Genetic Mutations and Somatic Anomalies in Association With 46,XY Gonadal Dysgenesis. Ferti Steril (2015) 103:1297–304. doi: 10.1016/j.fertnstert.2015.01.043 25813279

[53] Corbetta S Muzza M Avagliano L Bulfamante G Gaetti L Eller-Vainicher C. Gonadal Structures in a Fetus With Complete Androgen Insensitivity Syndrome and Persistent Müllerian Derivatives: Comparison With Normal Fetal Development. Ferti Steril (2011) 95:1119.e9–19.e14. doi: 10.1016/j.fertnstert.2010.09.028 20971460

[54] Valeri C Lovaisa MM Racine C Edelsztein NY Riggio M Giulianelli S. Molecular Mechanisms Underlying AMH Elevation in Hyperoestrogenic States in Males. Sci Rep (2020) 10:15062. doi: 10.1038/s41598-020-71675-7 32934281 PMC7492256

[55] Liu Q Yin X Li P. Clinical, Hormonal and Genetic Characteristics of Androgen Insensitivity Syndrome in 39 Chinese Patients. Reprod Biol Endocrinol (2020) 18:1–9. doi: 10.1186/s12958-020-00593-0 32345305 PMC7187512

[56] Guaragna-Filho G Calixto AR Astur A.B.L.D.V. Paula GB Oliveira LC Morcillo AM. Leydig and Sertoli Cell Function in Individuals With Genital Ambiguity, 46, XY Karyotype, Palpable Gonads and Normal Testosterone Secretion: A Case-Control Study. Sao Paulo Med J (2022) 140:163–70. doi: 10.1590/1516-3180.2021.0042.r1.08062021 PMC9610247 35137906

[57] Hellmann P Christiansen P Johannsen TH Main KM Duno M Juul A. Male Patients With Partial Androgen Insensitivity Syndrome: A Longitudinal Follow-Up of Growth, Reproductive Hormones and the Development of Gynaecomastia. Arch Dis Child (2012) 97:403–9. doi: 10.1136/archdischild-2011-300584 22412043

[58] Boisen K Kaleva M Main K Virtanen H Haavisto A Schmidt I. Difference in Prevalence of Congenital Cryptorchidism in Infants Between Two Nordic Countries. Lancet (2004) 363:1264–9. doi: 10.1016/S0140-6736(04)15998-9 15094270

[59] Hildorf S Dong L Thorup J Clasen-Linde E Andersen CY Cortes D. Sertoli Cell Number Correlates With Serum Inhibin B in Infant Cryptorchid Boys. Sexual Dev (2019) 13:74–82. doi: 10.1159/000497374 30889614

[60] Grinspon RP Gottlieb S Bedecarrás P Rey RA. Anti-Müllerian Hormone and Testicular Function in Prepubertal Boys With Cryptorchidism. Front Endocrinol (Lausanne) (2018) 9:182. doi: 10.3389/fendo.2018.00182 29922225 PMC5996917

[61] Lucas-Herald AK Kyriakou A Alimussina M Guaragna-Filho G Diver LA McGowan R. Serum Anti-Müllerian Hormone in the Prediction of Response to hCG Stimulation in Children With DSD. J Clin Endocrinol Metab (2020) 105:dgaa052. doi: 10.1210/clinem/dgaa052 32016383 PMC7096311

[62] Skakkebaek NE Rajpert-De Meyts E Main KM. Testicular Dysgenesis Syndrome: An Increasingly Common Developmental Disorder With Environmental Aspects. Hum Reprod (2001) 16:972–8. doi: 10.1093/humrep/16.5.972 11331648

[63] Hoei-Hansen CE Holm M Rajpert-De Meyts E Skakkebaek NE. Histological Evidence of Testicular Dysgenesis in Contralateral Biopsies From 218 Patients With Testicular Germ Cell Cancer. J Pathol (2003) 200:370–4. doi: 10.1002/path.1372 12845633

[64] van den Driesche S Kilcoyne KR Wagner I Rebourcet D Boyle A Mitchell R. Experimentally Induced Testicular Dysgenesis Syndrome Originates in the Masculinization Programming Window. JCI Insight (2017) 2:e91204. doi: 10.1172/jci.insight.91204 28352662 PMC5358493

[65] Hutchison GR Scott HM Walker M McKinnell C Ferrara D Mahood IK. Sertoli Cell Development and Function in an Animal Model of Testicular Dysgenesis Syndrome1. Biol Reprod (2008) 78:352–60. doi: 10.1095/biolreprod.107.064006 17928633

[66] Brauner R Neve M Allali S Trivin C Lottmann H Bashamboo A. Clinical, Biological and Genetic Analysis of Anorchia in 26 Boys. PLoS One (2011) 6:e23292. doi: 10.1371/journal.pone.0023292 21853106 PMC3154292

[67] Lin L Achermann J. Steroidogenic Factor-1 (SF-1, Ad4BP, NR5A1) and Disorders of Testis Development. Sexual Dev (2008) 2:200–9. doi: 10.1159/000152036 PMC2645687 18987494

[68] Werner R Mönig I Lünstedt R Wünsch L Thorns C Reiz B. New NR5A1 Mutations and Phenotypic Variations of Gonadal Dysgenesis. PLoS One (2017) 12:e0176720. doi: 10.1371/journal.pone.0176720 28459839 PMC5411087

[69] Allali S Muller J-B Brauner R Lourenço D Boudjenah R Karageorgou V. Mutation Analysis of NR5A1 Encoding Steroidogenic Factor 1 in 77 Patients With 46, XY Disorders of Sex Development (DSD) Including Hypospadias. PLoS One (2011) 6(10):e24117. doi: 10.1371/journal.pone.0024117 22028768 PMC3197579

[70] Matsumoto AM Karpas AE Bremner WJ. Chronic Human Chorionic Gonadotropin Admininstration in Normal Men: Evidence That Follicle-Stimulating Hormone Is Necessary for the Maintenance of Quatitatively Normal Spermatogenesis in Man. J Clin Endocrinol Metab (1986) 62:1184–92. doi: 10.1210/jcem-62-6-1184 3084535

[71] Tapanainen JS Aittomäki K Min J Vaskivuo T Huhtaniemi IT. Men Homozygous for an Inactivating Mutation of the Follicle-Stimulating Hormone (FSH) Receptor Gene Present Variable Suppression of Spermatogenesis and Fertility. Nat Genet (1997) 15:205–6. doi: 10.1038/ng0297-205 9020851

[72] Garg V Kathiriya IS Barnes R Schluterman MK King IN Butler CA. GATA4 Mutations Cause Human Congenital Heart Defects and Reveal an Interaction With TBX5. Nature (2003) 424:443–7. doi: 10.1038/nature01827 12845333

[73] Tevosian SG Albrecht KH Crispino JD Fujiwara Y Eicher EM Orkin SH. Gonadal Differentiation, Sex Determination and Normal Sry Expression in Mice Require Direct Interaction Between Transcription Partners GATA4 and FOG2. Development (2002) 129(19):4627–34. doi: 10.1242/dev.129.19.4627 12223418

[74] Lourenço D Brauner R Rybczyńska M Nihoul-Fékété C McElreavey K Bashamboo A. Loss-Of-Function Mutation in GATA4 Causes Anomalies of Human Testicular Development. Proc Natl Acad Sci (2011) 108:1597–602. doi: 10.1073/pnas.1010257108 PMC3029689 21220346

[75] Grinspon RP Loreti N Braslavsky D Valeri C Schteingart H Ballerini MG. Spreading the Clinical Window for Diagnosing Fetal-Onset Hypogonadism in Boys. Front Endocrinol (2014) 5. doi: 10.3389/fendo.2014.00051 PMC4019849 24847309

[76] Young J Chanson P Salenave S Noeül ML Brailly S O’Flaherty M. Testicular Anti-Müllerian Hormone Secretion Is Stimulated by Recombinant Human FSH in Patients With Congenital Hypogonadotropic Hypogonadism. J Clin Endocrinol Metab (2005) 90:724–8. doi: 10.1210/jc.2004-0542 15536161

[77] Kubini K Zachmann M Albers N Hiort O Bettendorf M Woülfle J. Basal Inhibin B and the Testosterone Response to Human Chorionic Gonadotropin Correlate in Prepubertal Boys1. J Clin Endocrinol Metab (2000) 85:134–8. doi: 10.1210/jc.85.1.134 10634376

